# Trust-Wide Re-Audit on Prescription of Medications in Children Under 18 With Autism Spectrum Disorder (ASD) in Child and Adolescent Mental Health Services (CAMHS) at Black Country Healthcare NHS Foundation Trust (BCHFT)

**DOI:** 10.1192/bjo.2026.11563

**Published:** 2026-06-30

**Authors:** Reka Ajay Sundhar, Laitan Adewunmi, Rashmi Vishwanath, Bolutife Oyatokun, Suma Ujrebail

**Affiliations:** 1 Black Country Healthcare NHS Foundation Trust, Dudley, United Kingdom; 2 Black Country Healthcare NHS Foundation Trust, Sandwell, United Kingdom; 3 Black Country Healthcare NHS Foundation Trust, Walsall, United Kingdom

## Abstract

**Aims:**

This comprehensive Trustwide Reaudit aimed to evaluate patterns of psychotropic and sleepmedication prescribing within the CAMHS service at Black Country Healthcare NHS Foundation Trust for children diagnosed with Autism Spectrum Disorder (ASD). It comparedthe current practice against NICE guidelines. The key objectives were to identify variations in prescribing practices across different localities within the Trust and to assess both the improvements achieved and the remaining gaps when comparing the initial audit with the reaudit.

**Methods:**

A detailed retrospective review was carried out on 137 randomly selected cases involving children diagnosed with ASD who had been prescribed psychotropic or sleep medications. Comprehensive examination of patient records, including progress notes and clinic letters, enabled the collection of robust and meaningful data. To minimise bias, cases from each locality were evaluated by clinicians from different areas within the Trust. The assessment focused on comparing prescribing practices against NICE guidelines, and all stages of the process were conducted in accordance with strict ethical standards

**Results:**

Across the cohort of 137 children diagnosed with ASD, several areas of clinical practice showed clear improvement compared with the initial audit as follows. The proportion of children receiving alternative interventions before starting medication increased from 44% to 60%, and documentation of consent rose markedly from 62% to 93%. Specialists continued to initiate psychotropic medications at the minimum effective dose, and timely followup within 3–4 weeks improved from 70% to 75%. Recording of side effects also increased, moving from 61% to 70%, while documentation of prescribing indications rose from 83% to 93%, reflecting stronger clinical rationale and overall enhancement in practice standards in the Re-Audit.

**Conclusion:**

The Reaudit showed marked progress in several key areas, including consent procedures, the use of prior interventions, followup practices, and overall documentation,when compared with the initial audit. However, despite these improvements, the findings revealed inconsistencies in some areas in adherence to NICE guidelines. To address these gaps in the future, recommendations were made to strengthen collaboration with supporting agencies to increase the use of nonpharmacological interventions before medication is considered. Additional recommendations included implementing trustwide clinic letter templates to standardise documentation of indications, side effects, and consent, and to establish consistent followup intervals for reviewing medication side effects. These steps were viewed as essential for ensuring safe, effective, and highquality patient care.

